# Identical wave forms of vena cava and pulmonary artery during pulmonary artery catheter insertion due to kinking: a case report of a rare complication

**DOI:** 10.1186/s40064-016-3050-3

**Published:** 2016-08-19

**Authors:** Masayuki Oshima, Tatsuki Onishi, Hitomi Iwata, Kazuyoshi Aoyama

**Affiliations:** 1 Department of Anesthesia, Kobari General Hospital, 29-1 Yokouchi, Noda, Chiba 278-0004 Japan; 2 Department of Anesthesia, Metropolitan Bokutoh Hospital, Tokyo, Japan; 3 Department of Anesthesia and Pain Medicine, Hospital for Sick Children, Toronto, Canada

**Keywords:** Pulmonary artery catheter, Knotting, Kinking, Fluoroscopic guidance

## Abstract

**Background:**

We experienced a rare malposition of a pulmonary artery catheter due to kinking in a 63-year-old male who was scheduled for an off-pump coronary artery by-pass graft.

**Findings:**

Given the difficulty to obtain stable pulmonary artery waveform, we discovered that the two waveforms of the distal and proximal ports of the pulmonary artery catheter were completely identical. Subsequent fluoroscopy revealed that because the catheter had formed a kink around the apex of the right ventricle, the distal port faced the proximal port of the catheter.

**Conclusions:**

We recommend that both ports of the pulmonary artery catheter need to be monitored simultaneously in case neither portable fluoroscopy nor transesophageal echo is available.

## Background

Complications involving pulmonary artery (PA) catheterization are varied and include arrhythmias, pneumothorax, intracardiac rupture, pulmonary hemorrhage, and bacteremia (Dieden et al. 1987). Intravascular devices such as catheters can kink, loop and even knot during insertion. Major complications including knot formation have been reported with an incidence of 0.1 % (Bossert et al. 2006). Despite relatively rare incidence, they have the potential to result in severe consequences.

Knotting of intravascular catheters was first reported in Johanson et al. (1954). Since then, PA catheters accounted for more than two thirds of the total reported cases of intravascular knotting (Georghiou et al. 2004). This may in part be due to their inherently soft design and long length that may render them vulnerable to twisting, curling and knot formation during insertion.

## Case report

We report a rare malposition of a PA catheter secondary to kinking in a 63-year-old male who was scheduled for an off-pump coronary artery by-pass graft. The patient had a history of diabetes mellitus with neuropathy, early stage nephropathy and retinopathy. The pre-operative echocardiogram was significant for diffuse left ventricular hypokinesis with a corresponding ejection fraction of 51 %. The right ventricle size was normal and there was no evidence of pulmonary hypertension. After placement of standard monitors and preoxygenation of the patient, midazolam, fentanyl and rocuronium were administered. The patient was then intubated and maintenance of anesthesia was achieved using 1 % sevoflurane along with a continuous infusion of remifentanil. Subsequently, an arterial line was placed and a central venous catheter and a sheath for a PA catheter were inserted in the right internal jugular vein. The patient showed stable hemodynamics throughout the insertion of the catheters.

**Fig. 1:**
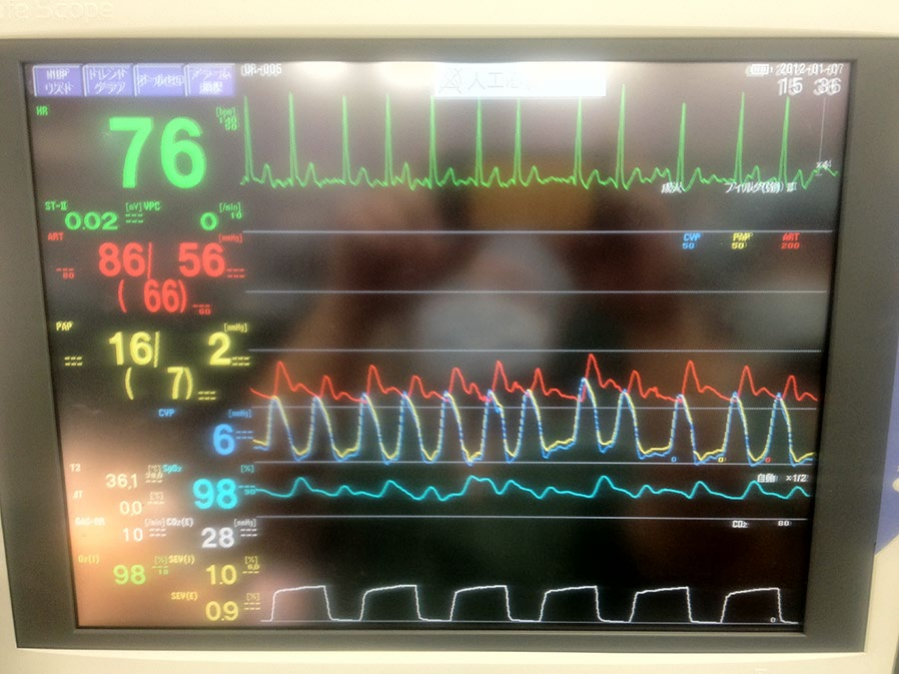
Distal port pressure (*yellow line*) and proximal port pressure (*blue line*) showing identical waveforms

During insertion of the PA catheter, we monitored the pressure waveform from the distal catheter port. The catheter was advanced to the right ventricle at a depth of 40 cm, then, was further advanced to the pulmonary artery at a depth of 55 cm. However, it was relatively easy to drop back the catheter to the right ventricle. Even after several attempts, no stable pulmonary artery waveform was obtained. We then discovered that the distal and proximal ports presented the same waveform pattern. We made the range of the two pressure lines the same and then realized that the two waveforms were completely identical (Fig. 1).

**Fig. 2:**
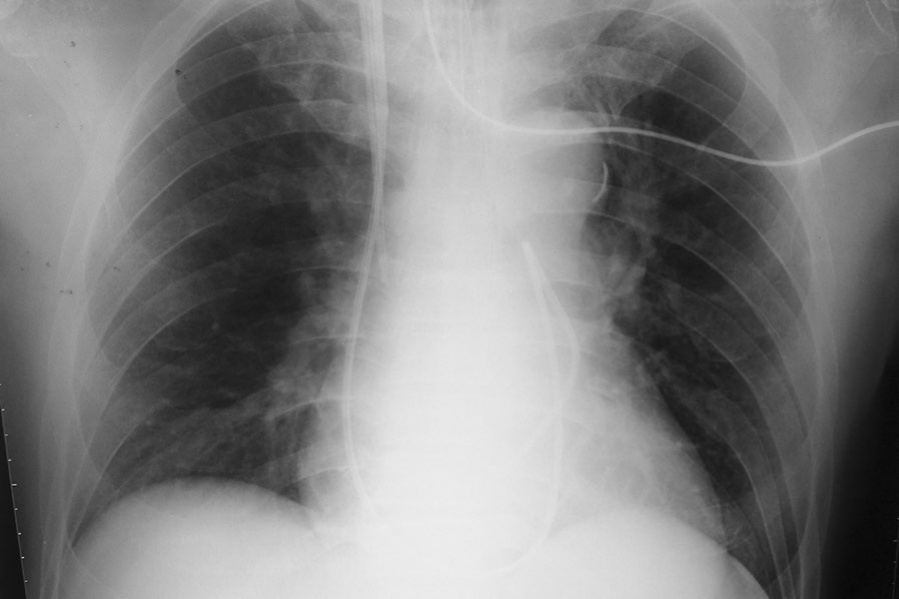
Chest X-ray showing kinked pulmonary catheter

**Fig. 3:**
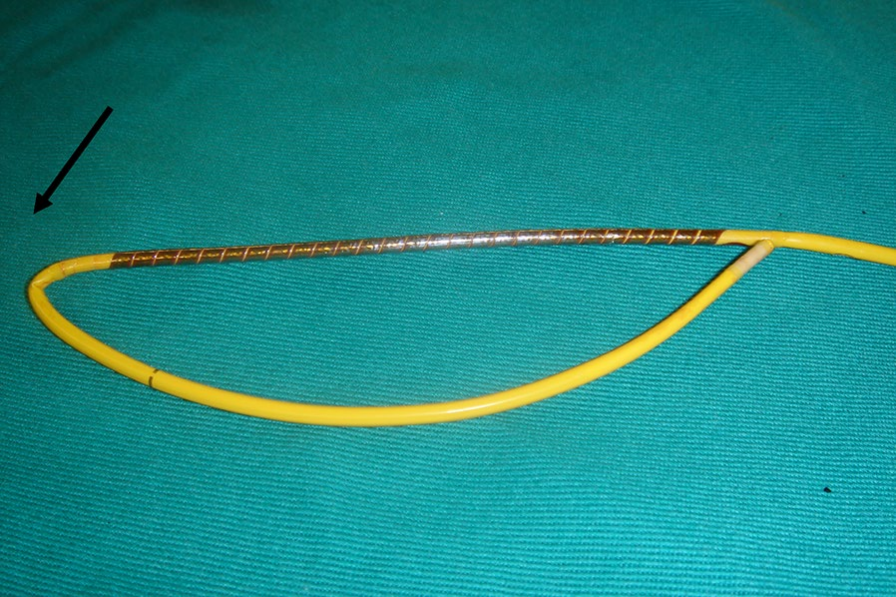
When bent at just distal site of thermo-dilution lead occurs, two pressure ports face each other (indicated by *arrows*)

We first suspected a mechanical problem with the catheter itself such as communication between lumens, so we flushed saline in each lumen. This changed each waveform independently, thus, communication between lumens seemed to be unlikely. Although we tried to remove the catheter, it was firmly in place at 15 cm depth. Flushing of cold saline through the distal port and placement of a guide wire were not successful in attempts to remove the catheter. We then utilized portable fluoroscopy to visualize the catheter in situ. We were able to determine that the catheter had formed a kink around the apex of the right ventricle (Fig. 2). With the use of a guide wire and alternating patient body position, we were able to straighten the catheter, which allowed for its subsequent removal. The removed catheter showed evidence of bend at the distal site of the thermo-dilution lead (Fig. 3). While reinsertion of new PA catheter, fluoroscopy showed that a kink started to form around the apex of the right ventricle. However, this time we advanced the PA catheter successfully using fluoroscopy and alternations in patient body position. Thereafter, the surgical procedure and postoperative course were both uneventful.

## Discussion

The anatomical complexity of the right heart can contribute to PA catheter kinking and looping. We experienced bend at the distal site of the catheter thermo-dilution lead. This may, in part, be due to right heart anatomy and due to the structural difference between the thermo-dilution lead and the rest of the catheter. In our situation, the distal port faced the proximal port of the catheter, which resulted in both ports displaying the same pressure waveform.

When anesthesiologists insert a PA catheter with two pressure lines (one for arterial pressure, the other for the distal port of a pulmonary artery catheter), they have no idea what the waveform at the proximal port shows. It is certainly important to keep close eyes on the depth of a PA catheter during the insertion so that we could consider possible kinking in early stage. However, the depth of a PA catheter is variable depending on where you insert the catheter and patient’s height (Tempe et al. 2006).

Following our experience, we decided to start monitoring the waveforms of both the distal and the proximal port during the insertion of a PA catheter. This will help detect the formation of catheter kinking in its early stage, and hopefully prevent its progression as kinking and looping are known to be precursors to catheter knotting. An alternative method to detect catheter kinking is to perform simultaneous transesophageal echo (TEE), to guide the advancement of the catheter. Yet, the downside of this approach is that it would require an experienced second individual to perform the echo. In addition, especially in circumstance with relatively limited resources compared to operating rooms (e.g. Intensive Care Units), it may not be feasible to access an TEE for an insertion of a PA catheter. This may be the case for portable fluoroscopy as well. Hence, simultaneous monitoring of distal and proximal ports of a PA catheter during the insertion is likely an easiest way to detect such a malposition of a PA catheter during the insertion, and recommended to follow in circumstances where neither TEE nor portable fluoroscopy is available.

## Conclusion

In summary, to avoid formation and development of a PA catheter kinking, we strongly recommend that both distal and proximal catheter ports need to be monitored simultaneously in addition to close eyes on the depth of a PA catheter during the insertion, especially in a setting neither portable fluoroscopy nor TEE is available.
